# Sequencing of a QTL-rich region of the *Theobroma cacao *genome using pooled BACs and the identification of trait specific candidate genes

**DOI:** 10.1186/1471-2164-12-379

**Published:** 2011-07-27

**Authors:** Frank A Feltus, Christopher A Saski, Keithanne Mockaitis, Niina Haiminen, Laxmi Parida, Zachary Smith, James Ford, Margaret E Staton, Stephen P Ficklin, Barbara P Blackmon, Chun-Huai Cheng, Raymond J Schnell, David N Kuhn, Juan-Carlos Motamayor

**Affiliations:** 1 Clemson University Genomics Institute, Clemson University, 51 New Cherry Street, Clemson, SC 29634, USA; 2 Department of Genetics & Biochemistry, Clemson University, 51 New Cherry Street, Clemson, SC 29634, USA; 3 Center for Genomics and Bioinformatics, Indiana University, 915 E. Third Street, Bloomington, IN 47405, USA; 4 IBM T.J. Watson Research Center, 1101 Kitchawan Road, Yorktown Heights, NY 10598, USA; 5 Subtropical Horticulture Research Station, USDA-ARS, 13601 Old Cutler Road, Miami, FL 33158, USA; 6 Mars Incorporated, 800 High Street, Hackettstown, NJ 07840, USA

**Keywords:** next-generation sequencing, QTL sequencing, fungal disease resistance, chocolate

## Abstract

**Background:**

BAC-based physical maps provide for sequencing across an entire genome or a selected sub-genomic region of biological interest. Such a region can be approached with next-generation whole-genome sequencing and assembly as if it were an independent small genome. Using the minimum tiling path as a guide, specific BAC clones representing the prioritized genomic interval are selected, pooled, and used to prepare a sequencing library.

**Results:**

This pooled BAC approach was taken to sequence and assemble a QTL-rich region, of ~3 Mbp and represented by twenty-seven BACs, on linkage group 5 of the *Theobroma cacao *cv. Matina 1-6 genome. Using various mixtures of read coverages from paired-end and linear 454 libraries, multiple assemblies of varied quality were generated. Quality was assessed by comparing the assembly of 454 reads with a subset of ten BACs individually sequenced and assembled using Sanger reads. A mixture of reads optimal for assembly was identified. We found, furthermore, that a quality assembly suitable for serving as a reference genome template could be obtained even with a reduced depth of sequencing coverage. Annotation of the resulting assembly revealed several genes potentially responsible for three *T. cacao *traits: black pod disease resistance, bean shape index, and pod weight.

**Conclusions:**

Our results, as with other pooled BAC sequencing reports, suggest that pooling portions of a minimum tiling path derived from a BAC-based physical map is an effective method to target sub-genomic regions for sequencing. While we focused on a single QTL region, other QTL regions of importance could be similarly sequenced allowing for biological discovery to take place before a high quality whole-genome assembly is completed.

## Background

For more than a decade, whole-genome sequencing strategies have typically employed one of two strategies: the BAC-by-BAC approach in which BAC clones that represent a minimum tiling path (MTP) are sequenced Sanger-style, as was taken for the rice and maize projects [1,2], or whole-genome shotgun (WGS) sequencing using random Sanger-style sequencing of entire genomic libraries of clones with varying insert size, such as was used to sequence the genomes of black cottonwood, grapevine, and sorghum [3-5]. Traditional *de novo *sequencing of large, complex eukaryotic genomes is plagued with assembly challenges caused by repetitive DNA and segmental duplications. Misassembly of distal genomic regions is always a potential pitfall, but this can be localized and minimized using a targeted sequencing approach including BAC-by-BAC sequencing.

Given the cost of a Sanger-sequence-based BAC-by-BAC approach, alternative techniques for targeting sub-genomic regions for sequencing are being explored that utilize the high sequencing depth achievable using next-generation sequencing technologies. For example, to determine if deep Roche/454 sequencing of pooled BAC clones effectively generated an accurate sub-genomic assembly, Rounsley *et al. *sequenced and assembled a 19 Mbp region of the short arm of chromosome 3 in rice; they concluded that assembly of six BAC pools, with an MTP derived from a physical map of approximately 3 Mbp, was accurate [6]. Using the 454 next-generation sequence reads, Rounsley *et al. *were able to assemble the 3 Mbp rice fragments with an N50 contig size ranging from 10.8 Kbp to 19.9 Kbp and an N50 scaffold size ranging from 243 Kbp to 518 Kbp. Other studies in barley [7], salmon [8], and melon [9] have been carried out using a similar BAC pooling and 454 sequencing strategy that allows for high quality sequencing of sub-genomic regions of high priority (e.g. QTL intervals or poorly resolved WGS assembly regions) at a cost far less than that of whole-genome sequencing.

*Theobroma cacao*, with its relatively small genome size (330-430 Mbp; [10-12]) and High Information Content Fingerprinting (HICF)-based [13] physical map (*see Saski et al companion paper*) that includes BAC-end sequences (BES), serves as an ideal test case for pooled-BAC sequencing. Reference sequences exist as the genomes of *T. cacao *cv. Criollo [10] and cv. Matina 1-6 http://www.cacaogenomedb.org have been sequenced. Multiple QTLs underlying traits such as fungal disease resistance have been identified and serve as important sequencing targets [14-24]. Of particular interest are regions that provide resistance to black pod, a disease caused by a fungal pathogen of mixed *Phytophthora *species [25,26]. Black pod decreases cacao yields by an estimated 20-30% annually [27]. Isolating genes responsible for resistance to black pod is of high importance to cacao breeding programs [28].

*T. cacao *HICF physical map contig 23, the subject of our study, is located on *T. cacao *linkage group 5 (LG5) and contains 15 microsatellite markers spanning 16 cM (based on a consensus map [29]). Three QTLs have been mapped to this region including a consensus QTL for black pod resistance (BP) and QTLs for two horticultural traits: bean shape index (BSI) and pod weight (PW) (Table 1). The BP QTLs were first identified by Risterucci *et al. *working with a population developed in Côte d'Ivoire [14]. Progeny were derived from a cross of a seedling of 'SCA6' crossed with an Upper Amazon Forastero clone known to contain resistance to BP, high productivity ('SCA'6x'H'), and a Trinitario variety. The male parent, 'IFC1,' is a highly homozygous Lower Amazon Forastero (Amelonado type) susceptible to BP. Progeny of this cross were evaluated using leaf-disc inoculation with three *Phytophthora *species, *P. megakarya*, *P. palmivora *and *P. capsici*, using two strains of each species. The original genetic map was made using AFLP markers and a map with 213 markers was produced; thirteen QTLs for BP resistance were reported all of which conveyed resistance to all three species [14]. This AFLP map was later augmented with microsatellite markers [24]. Using the microsatellite markers in the original progeny, common markers were then used to align the AFLP map with the consensus map ('UPA402' × 'UF676'). Thirteen consensus BP QTL were identified using a meta-analysis approach [30], two of which are on LG5 and one, cBP-12 QTL, is located on HICF contig 23 [24]. The cBP-12 QTL is located between 8.75 and 13.5 cM with the most significant peak at 11.1 cM and it explains 49.6% of the variation for this trait using a detached pod test [24]. QTLs for PW and BSI have also been located on the distal end of LG5 [15,16]. Both QTL co-locate with the cBP-12 QTL.

**Table 1:** QTLs localized to the *T.cacao *(cv. Matina 1-6) FPC contig 23 (LG5) sub-genome region

Trait Name	Mapping population	Max LOD	Map Position (cM)*	Most significant locus/peak (cM)	Phenotypic R2	Left flanking locus	Right flanking locus
Black pod 12 QTL (consensus)	(SCA6xH)xIFC1	3.9	8.75 to 13.5	11.1	49.6	mTcCIr265	gTcCIR139a
Bean shape index	S52xCatongo	5.3	0 to 17	6	17.1	gTcCIR148	cTcCIR73
Pod weight	S52xCatongo	4.8	0 to 17	6	13.8	gTcCIR148	AFLP_SxC39-1

*Map position from composite map (Brown et al. [29] or Lanaud et al. [24]).

Here, we describe and evaluate the reconstruction of this small QTL-rich, sub-genomic region (HICF contig 23) of *T. cacao *using a pooled BAC shotgun sequencing and assembly approach. This segment spans approximately 3 Mbp as estimated by the HICF physical map (*see Saski et al companion paper*). We sequenced the 27 BACs comprising the MTP as individual linear or pooled paired-end libraries using the 454 Titanium platform. The scaffolds obtained from a *de novo *assembly were ordered and oriented solely by mapping BES based on the known physical map MTP order. To assess the 454 assembly quality, we also sequenced 10 contiguous BACs from the MTP using Sanger sequencing. Once a quality assembly was constructed, candidate genes were mapped and putative genes conferring black pod resistance, bean shape index, and pod weight were identified as a first step in further evaluation. We also empirically estimated the minimum paired/linear 454 read coverage necessary to assemble high quality sub-genomic 3 Mbp regions. We discuss other practical details helpful for successfully sequencing high-priority genomic regions of similar size from any organism for which a physical map has been constructed.

## Results

### 454 Sequencing and Preprocessing

**Figure 1:**
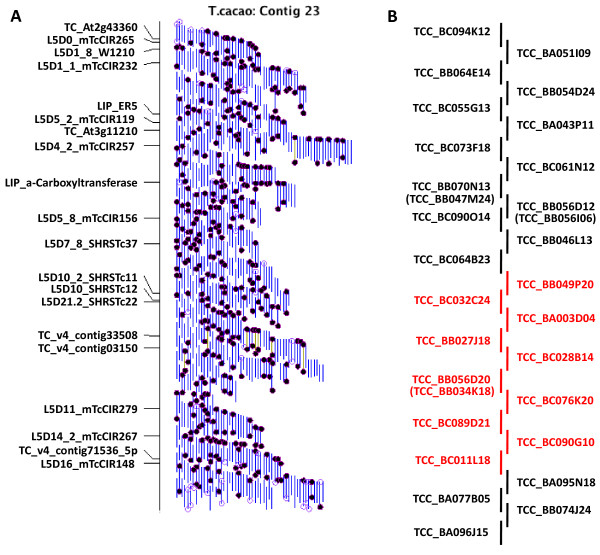
**Physical map and minimum tiling path selection for pooled BAC sequencing strategy**. **A**. Physical map of Contig 23. Blue lines represent BAC clones that cluster together, based on restriction profiles, forming Contig 23. Closed circles on clone ends represent BAC ends that were sequenced, open circles indicate that no BESs were available. The anchored genetic/STS markers are on the left and are ordered from distal to proximal chromosomal locations. **B**. A selected minimum tiling path of BACs for pooled sub-genome sequencing. Red BACs were sequenced using the Sanger method and used as high quality reference sequences. BACs in parentheses were alternate MTP BACs.

Pooled reads from twenty-seven *T. cacao *cv. Matina 1-6 BACs comprising the BP, BSI, and PW QTLs (Table 1) and HICF contig 23 MTP (Figure 1; *see Saski et al companion paper*) were obtained from a paired-end library preparation sequenced on one region of a 2-region 454 GS FLX Titanium PicoTiterPlate (PTP). Sequencing of a paired-end library using current technology yields both reads mated over the specified genomic jump distance, here called paired reads, and reads of unpaired genomic fragments, here called linear reads. Separate datasets from the sequencing of 27 individual shotgun-indexed (multiplex identifier (MID)-encoded, Roche/454 Sequencing) libraries were pooled and sequenced on a parallel region of the 2-region PTP. Raw paired and linear runs yielded 239.5 Mbp and 205.3 Mbp of sequence data, respectively (Table 2).

**Table 2:** 454 Read Pre-processing

Category	Paired	Linear
Titanium 454 Plates	0.5	0.5
Raw Reads (unbroken)	654,164	568,399
Raw Sequence (Mbp)	239.5	205.3
Average Raw Read Length (bp)	366.0	361.0
^1^Raw Coverage	79.8	68.4

***Stage I - Split Pairs***		

Reads	910,019	568,399
Sequence (Mbp)	201	205.3
Average Read Length (bp)	220.9	361.0
Stage I Coverage	67.0	68.4

***Stage II - Trim/Contamination Removal***		

Reads	496,756	338,957
Sequence (Mbp)	121.7	125.4
Average Read Length (bp)	244.9	367.9
Stage II Coverage	40.6	41.8

***Stage III - Mate Pair Detection***		

Reads	371,430	338,957
Sequence (Mbp)	67.3	125.4
Average Read Length (bp)	181.3	367.9
Stage III Coverage	22.4	41.8

*^1^Based on 3 Mbp sub-genome size*.

Raw reads from all sequencing reactions were extensively processed prior to assembly. After mate pair splitting, linker, bar-code, vector and *E. coli *contamination removal, coverages of the paired and pooled linear libraries were 22.4× and 41.8×, respectively (Table 2). Processed reads were then separated into pools containing linear (L), mate paired (PP), and no-mate pair singletons (NM), each pool comprising 5× coverage. The NM sequence set contains unpaired reads from the paired read library and was generated to determine if unmated reads can substitute for linear reads, which would reduce the need for the preparation of a costly second library.

### Assembly and Pseudomolecule Construction

A total of twenty-nine 454 assemblies representing HICF physical map contig 23 were obtained using the Celera wgs-assembler ([31]; CABOG v6.1). These assemblies were prepared using various combinations of 5×-increment read coverages of the L, PP, and NM pools of reads (see Table 3). In parallel, ten MTP BACs (~1 Mbp combined length) were individually sequenced using Sanger sequencing, individually assembled using Phrap [32], combined into a pseudomolecule, and used as a gold-standard reference sequence against which to assess the quality of the 454 assemblies.

**Table 3:** Basic assembly statistics for various read mixes

454 Read Mix^1^	Scaffolds *total*	Scaffolds *anchored*	Scaffold Length *(bp)*	Anchored Length *(%)*	Match Score^2 ^	Relocation Score^2^	Inversion Score^2 ^	Coverage Score^2^	Gap Length (*%N)*	Subgenome length (*bp)*
**35L-20PP**	**16**	**5**	**2,925,652**	**96.5%**	**0.64**	**1.00**	**1.00**	**0.93**	**0.05%**	**3,032,287**
20PP-20NM	23	5	2,948,378	95.6%	0.59	1.00	1.00	0.93	0.23%	3,085,172
10PP-10NM	20	9	2,919,380	96.1%	0.57	0.99	0.89	0.93	0.56%	3,037,466
20L-10PP	15	8	2,848,345	97.0%	0.56	1.00	1.00	0.92	0.88%	2,937,025
20L-15PP	14	7	2,894,304	96.7%	0.56	1.00	1.00	0.93	0.51%	2,993,053
20L-20PP	17	7	2,921,393	96.7%	0.56	1.00	1.00	0.93	0.30%	3,021,076
15PP-15NM	19	6	2,932,591	95.8%	0.55	1.00	1.00	0.93	0.39%	3,060,041
10L-20PP	13	7	2,917,670	96.6%	0.54	1.00	1.00	0.93	0.23%	3,021,711
15L-20PP	12	6	2,910,841	96.7%	0.54	1.00	1.00	0.93	0.29%	3,009,135
5L-20PP	15	6	2,874,297	94.5%	0.54	1.00	1.00	0.93	0.87%	3,040,453
10L-15PP	18	8	2,884,836	96.5%	0.53	1.00	1.00	0.92	0.74%	2,988,073
15L-15PP	15	8	2,892,073	96.7%	0.53	1.00	1.00	0.93	0.61%	2,989,876
5L-15PP	15	7	2,845,042	96.6%	0.53	1.00	1.00	0.92	1.48%	2,945,611
0L-20PP	13	6	2,877,053	94.9%	0.52	1.00	1.00	0.93	0.90%	3,031,950
10L-10PP	19	10	2,835,308	96.0%	0.52	1.00	1.00	0.91	1.02%	2,953,066
15L-10PP	16	8	2,835,593	96.8%	0.52	1.00	1.00	0.91	0.96%	2,928,679
20L-5PP	30	8	2,650,829	87.7%	0.52	0.99	0.94	0.72	2.84%	3,022,574
5L-10PP	25	10	2,734,631	96.2%	0.52	1.00	1.00	0.90	1.54%	2,843,359
0L-10PP	23	9	2,738,500	96.5%	0.51	0.93	0.78	0.90	1.68%	2,838,999
0L-15PP	17	7	2,836,521	94.5%	0.51	1.00	1.00	0.92	1.70%	3,001,136
10L-5PP	42	12	2,491,349	80.5%	0.51	0.89	0.38	0.66	5.20%	3,096,682
15L-5PP	34	9	2,588,122	84.3%	0.51	0.98	0.79	0.66	3.96%	3,068,882
5L-5PP	57	17	2,248,580	75.2%	0.51	0.91	0.62	0.66	7.06%	2,989,391
0L-5PP	57	17	2,214,995	74.7%	0.50	0.90	0.21	0.61	8.84%	2,967,162
35L-0PP	174	35	2,911,931	48.4%	0.50	0.99	0.28	0.51	0.00%	6,013,556
20L-0PP	70	26	1,812,591	59.4%	0.44	1.00	0.06	0.28	0.00%	3,048,991
10L-0PP	41	20	1,033,618	64.5%	0.40	1.00	0.55	0.17	0.00%	1,601,933
15L-0PP	57	23	1,485,809	59.2%	0.40	1.00	0.13	0.14	0.00%	2,510,749
5L-0PP	29	12	631,622	57.4%	0.27	1.00	0.12	0.07	0.00%	1,100,803

*^1^Number = fold sequencing depth; L = linear (shotgun) reads; PP = true pair reads; NM = Unmated (shotgun) reads*.

Table 3 summarizes characteristics of the various assemblies. For each assembly, scaffold order was determined in an automated fashion, with no manual editing, using the MTP BES information alone. BES-anchored scaffolds were then concatenated into pseudomolecules. In order to determine the most accurately assembled pseudomolecule representing HICF physical map contig 23, we aligned each sequence to the Sanger-sequenced gold standard reference pseudomolecule. Contiguity and length varied over a broad range (Table 3; additional file 1: Table S1). Match, relocation, inversion and coverage scores (ranging from 0 = worst to 1 = best) were determined (Table 3) as per a recently published scoring strategy [33], details of which are described in Methods. Many of the assemblies garnered good scores at lower sequence coverage mixes, a finding with implications for reduced sequencing costs. The optimal assembly was obtained with the 35L-20PP mix and consisted of 2.93 Mbp in which 96.5% of the scaffolds were anchored by MTP BES and 94.4% of the total BES were mapped (Tables 4 and 5; additional file 2: Table S2). Based on the missing 35L-20PP sequence relative to the Sanger pseudomolecule reference, we estimate by extrapolation that the actual sub-genomic region length is 3.03 Mbp (Table 3). The "missing" 102 Kbp of sequence could be in the form of unassembled degenerate contigs (contigs not placed in scaffolds) and surrogate contigs (those containing repetitive or ambiguous reads) (additional file 3: Table S3). It should be noted that while the 35L-20PP assembly was of the highest quality and was therefore chosen for further characterization, the assemblies that contained NM reads performed similarly well, indicating that a second library of linear reads may not be necessary (Table 3; additional file 4: Figure S1).

**Table 4:** MTP BAC Alignments to initial 35L-20PP Assembly

Scaffold ID	BAC	MTP Order	Scaffolds Hit	^1^BES Ends Mapped
scf7180000011013	TCC_BC094K12	0	1	2
scf7180000011013	TCC_BA051I09	1	1	2
scf7180000011013	TCC_BB064E14	2	1	2
scf7180000011013	TCC_BB054D24	3	1	2
scf7180000011013	TCC_BC055G13	4	1	2
scf7180000011013	TCC_BA043P11	5	1	2
scf7180000011013	TCC_BC073F18	6	1	2
scf7180000011013	TCC_BC061N12	7	1	2
scf7180000011009	TCC_BB070N13	8	1	2
n.a.	TCC_BB056D12	9	0	0
scf7180000011011	TCC_BC090O14	10	1	2
scf7180000011011	TCC_BB046L13	11	1	1
scf7180000011011	TCC_BC064B23	12	1	2
scf7180000011011	**TCC_BB049P20**	13	1	2
scf7180000011011	**TCC_BC032C24**	14	1	2
scf7180000011011	**TCC_BA003D04**	15	1	2
scf7180000011011	**TCC_BB027J18**	16	1	2
scf7180000011011	**TCC_BC028B14**	17	1	2
scf7180000011010	**TCC_BB056D20**	18	1	2
scf7180000011012	**TCC_BC076K20**	19	1	2
scf7180000011012	**TCC_BC089D21**	20	1	2
scf7180000011011	**TCC_BC090G10**	21	1	2
scf7180000011012	**TCC_BC011L18**	22	1	2
scf7180000011012	TCC_BA095N18	23	1	2
scf7180000011012	TCC_BA077B05	24	1	2
scf7180000011012	TCC_BB074J24	25	1	2
scf7180000011012	TCC_BA096J15	26	1	2

^1^94.4% BES Mapped; ^2^Bold = Sanger Sequenced BAC

**Table 5:** Pseudomolecule Scaffolds Anchored & Ordered by MTP

Assembly Mix	Scaffold	Length	Anchored^1^
35L-20PP	scf7180000011012	917,434	YES
35L-20PP	scf7180000011011	891,242	YES
35L-20PP	scf7180000011013	793,788	YES
35L-20PP	^2^scf7180000011009	116,402	YES
35L-20PP	^3^scf7180000011010	103,901	YES
35L-20PP	scf7180000011001	91,517	NO
35L-20PP	scf7180000011002	5,131	NO
35L-20PP	scf7180000011003	2,355	NO
35L-20PP	scf7180000011004	1,060	NO
35L-20PP	scf7180000011008	507	NO
35L-20PP	scf7180000010999	500	NO
35L-20PP	scf7180000011005	500	NO
35L-20PP	scf7180000011006	479	NO
35L-20PP	scf7180000011007	302	NO
35L-20PP	scf7180000010998	280	NO
35L-20PP	scf7180000011000	254	NO

^1^2,822,767 bp Anchored; 102,885 bp Unanchored; ^2^Replaced With TCC_BB047M24, TCC_BB055I06 Sanger Assembly; ^3^Replaced with TCC_BB034K18 Sanger assembly

Upon close inspection, it was noted that two 35L-20PP scaffolds each consisted of a single BAC, and that one BAC was missing from the assembly in that its BES did not align to any scaffold. Two of these BACs (TCC_BB056D12, TCC_BB070N13) were presumably mislabeled or the result of contamination from a neighboring well that occurred during or after construction of the physical map and we therefore pooled the wrong BAC for sequencing. Another MTP BAC may have resulted from an FPC assembly error (TCC_BB056D20), but its etiology is unclear. Three replacement MTP BACs (TCC_BB034K18, TCC_BB056I06, TCC_BB047M24) were therefore selected by searching flanking contig end sequence with HICF physical map contig 23 BAC-end sequences and selecting a BAC with paired-end sequences anchored to both contig ends. These alternate MTP BACS were individually Sanger-sequenced and substituted for the correct genomic sequence (Figure 1).

**Figure 2:**
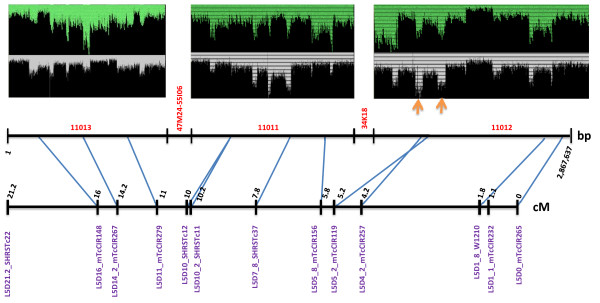
**Overview of 35L-10PP pooled BAC 454 assembly**. Read coverage in each scaffold is indicated in red. Arrows indicate points of BAC overlap. Black numbers are uncorrected pseudomolecule positions. Blue lines indicate genetic marker (in purple) positions in the pseudomolecule.

**Figure 3:**
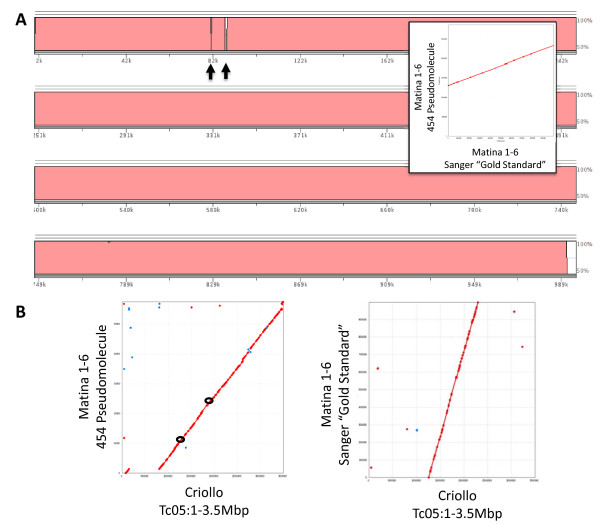
**Sanger-sequenced sub-region aligned to pooled BAC 454 assembly**. **A**. The Sanger-sequenced (reference) and 454 35L-20PP pseudomolecules are compared on this percent identity plot produced using the LAGAN algorithm [34] implemented using the VISTA browser [50]. Regions of assembly discrepancy are indicated with arrows. Inset shows MUMMER dot plot of this alignment. **B**. Left panel shows alignment of Matina 1-6 454 35L-20PP pseudomolecule with the first 3.5 Mbp of Tc05 contig derived from the *T. cacao *cv. Criollo genome assembly. Region between the black open circles indicate approximate location of Sanger-sequenced interval. The right panel shows alignment of Matina 1-6 Sanger-sequenced pseudomolecule against the same segment of the Criollo genome assembly.

The corrected 35L-20PP assembly was then validated by aligning it with genetic markers from the composite linkage map [29] localized to this region of the *T. cacao *genome (Figure 2). All markers were ordered correctly with the exception of one small inversion (Figure 2). Upon examination of high quality mate pairs and linear reads, read depth spikes of coverage where BACs overlap were apparent (Figure 2). Interestingly, although variation in stoichiometry as assumed by read coverage varied up to four-fold, these variations in coverage intensity appeared to have minimal effect on the overall assembly. Comparison of the 454 pseudomolecule to the Sanger reference using LAGAN [34] or MUMMER [35] indicated that a majority of the assembly in this region was consistent in that only six gaps between the two sequences were apparent (Figure 3A; additional file 5: Figure S2). Close inspection of these regions revealed that a gap in the 454 pseudomolecule was primarily due to regions of simple sequence repeats present in the Sanger-based assembly. Finally, we were able to localize the 454 pseudomolecule to the 'Tc05' contig from the recent release of the *T. cacao *cv. Criollo assembly [10]. A small inversion (~110 Kbp) and a large insertion (~654 Kbp) relative to the Criollo genome were observed (Figure 3B). It is unclear if these differences are due to polymorphism and/or misassembly. It should be noted that these comparisons were performed on the corrected pseudomolecule with the three Sanger BAC assemblies inserted into the pooled BAC 454 assembly. Therefore, while these comparisons validate the quality of the pseudomolecule assembly, they do not provide a pure comparison of 454 vs. Sanger-based assemblies.

### Annotating the assembly

We used publicly available *T. cacao *EST assemblies (NCBI UniGene Build#2) to annotate genes in the 35L-20PP pseudomolecule. From the set of 25,016 ESTs, we mapped 249 putative unigene clusters using moderate BLASTN [36] stringencies (additional file 6: Table S4). These unigenes were annotated for gene ontology [37], KEGG biochemical pathway [38], and conserved Interproscan protein domain [39] functional signatures. Annotations were compiled in a single row per unigene format (additional file 7: Table S5) or in a database-friendly format (additional file 8: Table S6). Unigene sequences are provided (additional file 9).

Descriptions of homologous genes and all individual annotations were manually inspected for relevance to the BP resistance, PW, and BSI traits. In the case of BP resistance, candidate resistance genes were selected by searching for unigenes associated with "stress," which narrowed the candidate black pod resistance genes from all 249 to 25 (Table 6). The specific terms used for selection were: "stress response to abiotic stimulus (GO:0009628);" "response to biotic stimulus (GO:0009607);" "response to endogenous stimulus (GO:0009719);" "response to extracellular stimulus (GO:0009991);" and "response to stress (GO:0006950)." In addition, gene annotations were inspected for relevance to the BSI and PW traits without strict criteria.

**Table 6:** Candidate resistance genes in Black Pod (BP) resistance QTL region

UnigeneID	Blast Hit Description	Accession	Instances	Method	Term Description
gnl_UG_Tcc_S51555371	heat shock	EC:1.3.1.74	1	Blast2GO	2-alkenal reductase
		GO:0000166	1	Blast2GO	nucleotide binding
		**GO:0006950**	**1**	**Blast2GO**	**response to stress**
		IPR019651	2	Interproscan	Glutamate dehydrogenase, NAD-specific
		none	1	Interproscan	SignalP
gnl_UG_Tcc_S51563205	fructose-bisphosphatase precursor	EC:1.3.1.74	1	Blast2GO	2-alkenal reductase
		EC:3.1.3.11	1	Blast2GO	fructose-bisphosphatase
		GO:0005576	1	Blast2GO	extracellular region
		GO:0005975	1	Blast2GO	carbohydrate metabolic process
		GO:0006091	1	Blast2GO	generation of precursor metabolites and energy
		**GO:0006950**	**1**	**Blast2GO**	**response to stress**
		GO:0009536	1	Blast2GO	plastid
		**GO:0009628**	**1**	**Blast2GO**	**response to abiotic stimulus**
		GO:0015979	1	Blast2GO	photosynthesis
		GO:0016787	1	Blast2GO	hydrolase activity
gnl_UG_Tcc_S51573205	probable plasma membrane intrinsic protein 1c	GO:0005215	1	Blast2GO	transporter activity
		GO:0005886	1	Blast2GO	plasma membrane
		GO:0006810	1	Blast2GO	transport
		**GO:0006950**	**1**	**Blast2GO**	**response to stress**
		GO:0009536	1	Blast2GO	plastid
		**GO:0009628**	**1**	**Blast2GO**	**response to abiotic stimulus**
		GO:0016020	1	Blast2GO	membrane
gnl_UG_Tcc_S51574569	at3g51780 orf3	GO:0000003	1	Blast2GO	reproduction
		**GO:0006950**	**1**	**Blast2GO**	**response to stress**
		GO:0008219	1	Blast2GO	cell death
		**GO:0009628**	**1**	**Blast2GO**	**response to abiotic stimulus**
		GO:0009791	1	Blast2GO	post-embryonic development
		IPR000626	2	Interproscan	Ubiquitin
		IPR003103	3	Interproscan	Apoptosis regulator, Bcl-2 protein, BAG
		IPR019955	2	Interproscan	Ubiquitin supergroup
gnl_UG_Tcc_S51576386	abc transporter family protein	EC:3.6.3.28	1	Blast2GO	phosphonate-transporting ATPase
		GO:0000166	1	Blast2GO	nucleotide binding
		GO:0005215	1	Blast2GO	transporter activity
		GO:0006810	1	Blast2GO	transport
		**GO:0009607**	**1**	**Blast2GO**	**response to biotic stimulus**
		GO:0016020	1	Blast2GO	membrane
		GO:0016787	1	Blast2GO	hydrolase activity
		IPR013525	2	Interproscan	ABC-2 type transporter;GO:0016020
gnl_UG_Tcc_S51582115	at3g51780 orf3	GO:0000003	1	Blast2GO	reproduction
		**GO:0006950**	**1**	**Blast2GO**	**response to stress**
		GO:0008219	1	Blast2GO	cell death
		**GO:0009628**	**1**	**Blast2GO**	**response to abiotic stimulus**
		GO:0009791	1	Blast2GO	post-embryonic development
gnl_UG_Tcc_S51583076	at5g01500 f7a7_20	GO:0005215	1	Blast2GO	transporter activity
		GO:0005739	1	Blast2GO	mitochondrion
		GO:0006091	1	Blast2GO	generation of precursor metabolites and energy
		GO:0006810	1	Blast2GO	transport
		GO:0009536	1	Blast2GO	plastid
		GO:0009579	1	Blast2GO	thylakoid
		**GO:0009607**	**1**	**Blast2GO**	**response to biotic stimulus**
		**GO:0009628**	**1**	**Blast2GO**	**response to abiotic stimulus**
		GO:0015979	1	Blast2GO	photosynthesis
		GO:0016020	1	Blast2GO	membrane
		GO:0019538	1	Blast2GO	protein metabolic process
		IPR018108	3	Interproscan	Mitochondrial substrate/solute carrier
		IPR023395	3	Interproscan	Mitochondrial carrier domain
gnl_UG_Tcc_S51585933	pseudo response regulator	GO:0003677	1	Blast2GO	DNA binding
		GO:0004871	1	Blast2GO	signal transducer activity
		GO:0005634	1	Blast2GO	nucleus
		GO:0005739	1	Blast2GO	mitochondrion
		GO:0006350	1	Blast2GO	transcription
		GO:0007165	1	Blast2GO	signal transduction
		**GO:0009628**	**1**	**Blast2GO**	**response to abiotic stimulus**
		GO:0016301	1	Blast2GO	kinase activity
		GO:0030528	1	Blast2GO	transcription regulator activity
gnl_UG_Tcc_S51595021	ammonium transporter	GO:0005215	1	Blast2GO	transporter activity
		GO:0005886	1	Blast2GO	plasma membrane
		GO:0006810	1	Blast2GO	transport
		**GO:0009607**	**1**	**Blast2GO**	**response to biotic stimulus**
		GO:0016020	1	Blast2GO	membrane
		IPR001905	6	Interproscan	Ammonium transporter
gnl_UG_Tcc_S51598545	asymmetric leaves1 and rough	GO:0003700	1	Blast2GO	sequence-specific DNA binding transcription factor activity
		GO:0005634	1	Blast2GO	nucleus
		GO:0006350	1	Blast2GO	transcription
		**GO:0006950**	**1**	**Blast2GO**	**response to stress**
		GO:0007275	1	Blast2GO	multicellular organismal development
		**GO:0009607**	**1**	**Blast2GO**	**response to biotic stimulus**
		**GO:0009628**	**1**	**Blast2GO**	**response to abiotic stimulus**
		GO:0009653	1	Blast2GO	anatomical structure morphogenesis
		**GO:0009719**	**1**	**Blast2GO**	**response to endogenous stimulus**
gnl_UG_Tcc_S51616674	3-oxo-5-alpha-steroid 4-dehydrogenase	EC:1.3.99.5	1	Blast2GO	3-oxo-5-alpha-steroid 4-dehydrogenase
		GO:0005737	1	Blast2GO	cytoplasm
		GO:0006629	1	Blast2GO	lipid metabolic process
		**GO:0009628**	**1**	**Blast2GO**	**response to abiotic stimulus**
		GO:0016020	1	Blast2GO	membrane
gnl_UG_Tcc_S51619644	**fungal defense**	none	0		
gnl_UG_Tcc_S51619902	sulfolipid synthase	GO:0006629	1	Blast2GO	lipid metabolic process
		**GO:0006950**	**1**	**Blast2GO**	**response to stress**
		GO:0007154	1	Blast2GO	cell communication
		GO:0009536	1	Blast2GO	plastid
		**GO:0009991**	**1**	**Blast2GO**	**response to extracellular stimulus**
		GO:0016740	1	Blast2GO	transferase activity
gnl_UG_Tcc_S51633831	heat shock	EC:1.3.1.74	1	Blast2GO	2-alkenal reductase
		GO:0000166	1	Blast2GO	nucleotide binding
		**GO:0006950**	**1**	**Blast2GO**	**response to stress**
		IPR019651	2	Interproscan	Glutamate dehydrogenase, NAD-specific
gnl_UG_Tcc_S51634817	thaumatin-like protein	GO:0005737	1	Blast2GO	cytoplasm
		**GO:0009607**	**1**	**Blast2GO**	**response to biotic stimulus**
		IPR001938	5	Interproscan	Thaumatin, pathogenesis-related
gnl_UG_Tcc_S51639853	gdp-mannose pyrophosphorylase	EC:2.7.7.13	1	Blast2GO	mannose-1-phosphate guanylyltransferase
		EC:2.7.7.22	1	Blast2GO	mannose-1-phosphate guanylyltransferase (GDP)
		GO:0005739	1	Blast2GO	mitochondrion
		GO:0005975	1	Blast2GO	carbohydrate metabolic process
		**GO:0006950**	**1**	**Blast2GO**	**response to stress**
		**GO:0009607**	**1**	**Blast2GO**	**response to biotic stimulus**
		**GO:0009628**	**1**	**Blast2GO**	**response to abiotic stimulus**
		**GO:0009719**	**1**	**Blast2GO**	**response to endogenous stimulus**
		GO:0016740	1	Blast2GO	transferase activity
		IPR001451	2	Interproscan	Bacterial transferase hexapeptide repeat
		IPR011004	2	Interproscan	Trimeric LpxA-like
		IPR018357	2	Interproscan	Hexapeptide transferase, conserved site
gnl_UG_Tcc_S51640638	at5g05440 k18i23_25	GO:0004872	1	Blast2GO	receptor activity
		GO:0005634	1	Blast2GO	nucleus
		GO:0005737	1	Blast2GO	cytoplasm
		GO:0007165	1	Blast2GO	signal transduction
		GO:0008289	1	Blast2GO	lipid binding
		**GO:0009719**	**1**	**Blast2GO**	**response to endogenous stimulus**
gnl_UG_Tcc_S51641476	dna binding protein	EC:2.7.11.17	1	Blast2GO	calcium/calmodulin-dependent protein kinase
		GO:0003677	1	Blast2GO	DNA binding
		GO:0004518	1	Blast2GO	nuclease activity
		GO:0006464	1	Blast2GO	protein modification process
		**GO:0006950**	**1**	**Blast2GO**	**response to stress**
		GO:0009536	1	Blast2GO	plastid
		GO:0016301	1	Blast2GO	kinase activity
gnl_UG_Tcc_S51660381	heat shock	EC:1.3.1.74	1	Blast2GO	2-alkenal reductase
		GO:0000166	1	Blast2GO	nucleotide binding
		**GO:0006950**	**1**	**Blast2GO**	**response to stress**
		IPR001023	2	Interproscan	Heat shock protein Hsp70
gnl_UG_Tcc_S51662116	ca2+ antiporter cation exchanger	GO:0005215	1	Blast2GO	transporter activity
		GO:0005773	1	Blast2GO	vacuole
		GO:0006810	1	Blast2GO	transport
		**GO:0006950**	**1**	**Blast2GO**	**response to stress**
		**GO:0009607**	**1**	**Blast2GO**	**response to biotic stimulus**
		**GO:0009628**	**1**	**Blast2GO**	**response to abiotic stimulus**
		GO:0016020	1	Blast2GO	membrane
		GO:0019725	1	Blast2GO	cellular homeostasis
		IPR004837	3	Interproscan	Sodium/calcium exchanger membrane region
gnl_UG_Tcc_S51666712	white-brown-complex abc transporter family	EC:3.6.3.28	1	Blast2GO	phosphonate-transporting ATPase
		GO:0000166	1	Blast2GO	nucleotide binding
		GO:0005215	1	Blast2GO	transporter activity
		GO:0006810	1	Blast2GO	transport
		**GO:0009607**	**1**	**Blast2GO**	**response to biotic stimulus**
		GO:0016020	1	Blast2GO	membrane
		GO:0016787	1	Blast2GO	hydrolase activity
		IPR003439	3	Interproscan	ABC transporter-like
		IPR017871	3	Interproscan	ABC transporter, conserved site
gnl_UG_Tcc_S51667842	sodium-and lithium-tolerant 1	**GO:0006950**	**1**	**Blast2GO**	**response to stress**
		**GO:0009628**	**1**	**Blast2GO**	**response to abiotic stimulus**
gnl_UG_Tcc_S51688650	type i small heat shock protein kda isoform	GO:0005737	1	Blast2GO	cytoplasm
		**GO:0006950**	**1**	**Blast2GO**	**response to stress**
		IPR002068	3	Interproscan	Heat shock protein Hsp20
		IPR008978	2	Interproscan	HSP20-like chaperone
gnl_UG_Tcc_S51695094	at3g53990 f5k20_290	**GO:0006950**	**1**	**Blast2GO**	**response to stress**
		**GO:0009628**	**1**	**Blast2GO**	**response to abiotic stimulus**
gnl_UG_Tcc_S51700457	pyruvate decarboxylase-1	EC:4.1.1.1	1	Blast2GO	pyruvate decarboxylase
		**GO:0006950**	**1**	**Blast2GO**	**response to stress**
		GO:0016020	1	Blast2GO	membrane
		GO:0016740	1	Blast2GO	transferase activity
		IPR012001	2	Interproscan	Thiamine pyrophosphate enzyme, N-terminal TPP-binding domain

The following **bold **terms were used for candidate gene selection: response to abiotic stimulus(GO:0009628); response to biotic stimulus(GO:0009607); response to endogenous stimulus(GO:0009719); response to extracellular stimulus(GO:0009991); response to stress(GO:0006950).

The following terms were omitted due to low information content: biological_process(GO:0008150); biosynthetic process(GO:0009058); binding(GO:0005488); catalytic activity(GO:0003824);cellular process(GO:0009987); DNA metabolic process(GO:0006259); metabolic process(GO:0008152). protein binding(GO:0005515).

## Discussion

We have used next-generation sequencing and a pooled MTP BAC approach to re-construct a high-quality 3 Mbp region of the *T. cacao *genome that contains genes putatively responsible for heritable resistance to the black pod fungal pathogen as well as genes associated with two horticultural traits: bean shape index and pod weight. Targeted sub-genome sequencing using a pooled BAC approach as described in this and other studies [7-9] is an alternative to whole-genome shotgun (WGS) sequencing that offers key relative advantages including fast sample to pseudomolecule processing time, reduced cost, and fewer misassemblies of distal chromosomal segments. This technique is especially useful when quality sequence from a specific genomic region of high importance, *e.g*. a QTL associated with a large phenotypic effect, is needed as a reference for localized functional genomics studies (genomic re-sequencing, expression microarray design, RNAseq, etc.). While many diploid genomes are rapidly becoming available using WGS techniques, especially large, complex, or polyploid genomes still pose challenges for accurate and cost-effective WGS. The investment in a complete physical map for the purpose of MTP discovery can accelerate assembly and improve accuracy in the *de novo *construction of high priority genome segments.

During this study, we encountered several issues of practical concern for researchers carrying out similar projects. First, the selection of an accurate MTP is paramount. As with many high-throughput genomics studies, the large number of 384-well plates needed for a 10×+ coverage BAC library increases the risk of mislabeling or inverting labels. In addition to human error, the limitations of fingerprinting algorithms to correctly assemble clones with extremely dense banding patterns due to over-representation of the restriction sites or clones that contain highly repetitive sequences can lead to statistical errors in selections of an MTP. In this study, we individually re-sequenced the misplaced BACs that we discovered, after assembly, had been incorrectly selected as part of the MTP. A re-fingerprinting of the MTP prior to library construction would ensure its accuracy prior to pooling. Second, creating a Sanger reference sequence from a subset of the MTP targeted for pooling enables testing for accuracy of assemblies comprised of various read mixtures (Table 3). While the optimal assembly with the 35L-20PP mixture was derived from all pre-processed reads, our data suggest that sequencing the pool at a lower coverage would result in only a minor sacrifice in assembly quality (Table 3). For example, it appears that unmated reads obtained from a paired-end library (NM reads) could substitute for a second linear library, at least with the Titanium platform (Table 3, additional file 1: Figure S1). Circumventing the construction and sequencing of a second linear library would be a significant cost advantage. Our study could be used as a baseline for determining sequencing depth in future pooled BAC *de novo *assembly experiments. However, as sequencing technologies improve critical factors such as read length, it would be prudent to reassess minimal coverage required for accurate assembly.

We used a *T. cacao *unigene set to identify potential genes in our *de novo*-assembled genome fragment containing the black pod resistance QTL. Future studies utilizing the *T. cacao *genome sequence ([10]; http://www.cacaogenomedb.org) should provide gene sets of higher quality. Of the 25,016 unique unigene clusters we utilized, 249 mapped to within the 3 Mbp pseudomolecule. After functional profiling was performed and annotations examined for the candidate unigenes, 25 unigenes were selected based on having annotations associated with biotic/abiotic stress responses (Table 6, entries in bold). The complete, fully annotated gene list can be found in additional file 7: Table S5. Eight of the 25 unigenes were annotated for "response to biotic stimulus (GO:0009607)" and one of these eight, gnl_UG_Tcc_S51634817, is especially intriguing. This unigene maps to the pseudomolecule as a single high-scoring segment pair (HSP) from position 1,349,423 to 1,349,927 (98.4% identity; BLASTN E-value = 0) and codes for a thaumatin-like protein which is part of the pathogenesis-related (PR) family of proteins implicated in systemic acquired and induced resistance mechanisms [40]. Members of this family have been shown to have antifungal activity specifically against *Phytophthora *spp. [41-43], the causal pathogens in black pod disease [26]. Another interesting gene, gnl_UG_Tcc_S51619644, mapped to the pseudomolecule as a single HSP from position 259,893 to 260,322 (99.5% identity; BLASTN E-value = 0). This unigene has homology to six Arabidopsis genes encoding "barley mildew resistance locus O (MLO)" proteins; members of this protein family, AT3G45290, AT1G11310, AT2G39200, AT2G17480, AT1G42560, and AT2G33670 (E-value range 9.4e-05 to 1.7e-10), contain seven transmembrane domains. MLO proteins have been shown to have antifungal properties and require a syntaxin, a glycosyl hydrolase and an ABC transporter to confer resistance through inhibition of cell entry [44]. While no gene associated with syntaxin function was identified in the black pod resistance region, two genes encoding ABC transporter activity were identified, gnl_UG_Tcc_S51576386, gnl_UG_Tcc_S51666712, and another gene in the region putatively encodes alpha 1,4-glycosyltransferase (IPR007652). These data do not prove a causal relationship between these putative genes and the black pod resistance trait; these genes are, however, logical candidates for genetic validation experiments.

We also searched for candidate genes underlying the bean shape index and pod weight QTLs also localized to this sub-genomic region. A single unigene with homology to alpha-expansin, gnl_UG_Tcc_S51616677, mapped to the pseudomolecule as 4 HSPs (1,216,177-1,216,492; 1,216,706-1,217,048; 1,217,733-1,218,053; 1,218,268-1,218,447). Alpha-expansins are involved in cell extension [45] and this gene could be involved in the BSI and/or PW phenotypes via pod/bean development processes. Another interesting unigene identified in the region encodes a POX-domain (IPR006563) and was detected as a single HSP (gnl_UG_Tcc_S51641876; 1,614,642-1,615,113) with homology to AT5G02030.1 (BLASTX; E-value = 1e-15), a member of the "three amino acid loop extension" (TALE) homeodomain superfamily; this superfamily has been associated with multiple phenotypes including silique development [46]. Finally, a single unigene, gnl_UG_Tcc_S51638298 (2 HSPs: 2,289,522-2,289,781; 2,289,950-2,290,371), encoding a Myb-domain (IPR014778) and a second unigene, gnl_UG_Tcc_S51592994 (2 HSPs: 1,994,206-1,994,694 and 1,995,074-1,995,265), share homology with a transcription factor, GT-3a, containing a MYB-like (IPR017877) DNA-binding domain. As with the gene candidates for black pod resistance, speculation that functions of these genes underlie variations in bean shape and pod weight present them as logical targets for genetic validation studies.

## Conclusions

In this study we efficiently and successfully sequenced a region of the *T. cacao *genome containing important QTLs for resistance to black pod and development of cacao fruit. We also identified candidate genes that may influence these traits. Our results suggest that pooling portions of a minimum tiling path derived from a BAC-based physical map is an effective method for identifying candidate genes contained within QTL intervals. In addition, our assembly is a high-quality reference sequence for mapping other reads resulting from next-generation sequencing applications to detect both DNA polymorphisms and differential gene expression patterns associated with the QTL region.

While we focused on a single QTL region, any QTL regions of special interest from any genome can be similarly sequenced thus allowing for timely discoveries of biological importance while a complete genome assembly of high quality is being constructed over a longer time frame. Our study suggests improvements that can be made in the practical aspects associated with pooled BAC sequencing and partial assembly of a genome. These details are important in planning a cost-effective sequencing strategy. For example, our results suggest that a single paired-end 454 library sequenced on one-half of a Titanium 454 plate may be sufficient to accurately assemble a 3 Mbp pool. We also found that one or two BACs sequenced using Sanger techniques serve as an excellent control when assessing assembly accuracy, information that will only become more critical as pool sizes increase.

## Methods

### HICF physical map contig and MTP selection

A detailed description of the HICF-based *T. cacao *physical map construction procedure can be found in (under review). Selection of the QTL-rich region was performed by localization of relevant genetic markers from LG5 onto BAC contig 23 of the physical map by DNA hybridization. Minimum tile path (MTP) BACs were selected using the MTP function internal of FPC [47] with the following parameters: 'Min FPC Overlap -15, Max FPC Overlap 50, and FromEnd 57'. A total of 27 BACs representing contig 23 were selected as a minimum tiling path and used for pooled, sub-genome sequencing.

### Sanger sequencing and assembly of 10 contiguous MTP BACs

The nucleotide sequences of the selected BACs were determined using the bridging shotgun method [48]. BAC DNA was extracted in midiprep quantities following manufacturer protocols (Qiagen, Valencia, CA) and subjected to random fragmentation with the HydroShear device (via Digilab, Holliston, Massachusetts) using the following parameters and a small shearing assembly unit: speed code 13, 20 cycles. Resulting DNA fragments were subject to end repair and phosphorylation. DNA fractions between 3.0-5.0 kb were resolved by agarose gel electrophoresis, eluted and ligated into the vector pBLUESCRIPT IIKS+ (Stratagene). The libraries were plated (using standard methods) and then arrayed into 10, 96-well microtiter plates for the sequencing reactions. Sequencing was performed using the BigDye Terminator v3.1 Cycle Sequencing Kit (Applied Biosystems, Carlsbad, CA). Sequence data from the forward and reverse priming sites of the shotgun clones were accumulated to an estimated 10× coverage, assuming a 135 kb average insert size, and assembled using the Phred-Phrap programs [32]. When gaps existed between contigs, custom oligos were designed from both ends flanking the gap using the primer design function internal to Consed [48] and gap-spanning sub-clones selected as templates for custom sequencing reactions. The final sequences were finished according to the Bermuda standards of finishing (< 1 error per 10 kb; and each base > phred30; http://www.genome.gov/10001812). These BACs were deposited into GenBank under the accession numbers: JN127762 - JN127775.

### Preparation of shotgun 454-sequencing libraries

DNA samples were quantified using Quant-iT Picogreen dsDNA Reagent (Invitrogen). A 5 μg aliquot of each BAC DNA preparation was nebulized at 43 psi for 1 minute and purified using a single Minelute column (Qiagen). DNA fragments were polished and adapted according to the GS Titanium General Library Preparation Kit (Roche/454 Sequencing) with the following exceptions. Enzymatic reactions were performed using half-scale volumes and incubation conditions for the end-polishing reactions were as follows: 12°C, 15 min.; 25°C, 15 min.; 70°C, 15 min. Polishing reactions were purified using 1.8× volume Agencourt AMPure SPRI beads (Beckman Coulter Genomics) and the polished fragments were eluted in 5 μl 10 mM Tris, pH 8.5. Ligations were performed in reaction volumes of 20 μls using Roche/454 MID adaptors 1-28; volumes were then increased to 50 μl with 30 μl 10 mM Tris, pH 8.5. Excess adaptors were removed using 0.6× volume SPRI beads and libraries were eluted in 10 μl 10 mM Tris, pH 8.5. Half-scale fill-in reactions were performed according to the manufacturer (Roche/454 Sequencing) and then volumes were increased to 50 μl with 25 μl 10 mM Tris, pH 8.5. Excess adaptors were removed using 0.6× volume SPRI beads and libraries were eluted in 15 μl 10 mM Tris, pH 8.5. AB adaptor fragment enrichment was not performed. The completed libraries were assessed on a Bioanalyzer (Agilent) using DNA High Sensitivity Chips and quantified as described above. An equimolar pool of the 27 libraries was prepared for sequencing. Emulsion PCR was performed for enrichment titration and sequencing according to the manufacturer (Roche/454 Sequencing). Titanium sequencing was performed on 1 region of a 2-region PicoTiterPlate (PTP). The NCBI SRA accession number for the linear BAC shotgun data is SRA027324.

### Preparation of paired-end 454-sequencing library

An equimolar pool of the 27 BAC preparations, totaling 6 μg, was prepared based on concentrations as determined above. This pool was sheared into approximately 3 kb fragments with a Digilab HydroShear (Genomic Solutions) using 10 cycles at speed code 16 followed by 30 cycles at speed code 13. A paired-end library set was prepared, according to the Roche/454 Sequencing 3 kb Paired End Library Preparation Manual, with six circularization reactions and two post-circularization amplifications each for a total of 12 sublibraries. Migration on Bioanalyzer mRNA Pico chips (Agilent) showed final ssDNA sublibrary lengths ranged from 473 nt to 600 nt. Sublibraries were quantified using Quant-iT OliGreen (Invitrogen) and pooled before sequencing. Sequencing was performed on 1 region of a 2-region PTP as described above. The NCBI SRA accession numbers for the 3 kb paired data of the BAC pool is SRA027323.

### Pooled BAC assembly and 454 pseudomolecule construction

Raw 454 reads from a single Titanium run (above) were split using the Celera CABOG Assembler v6.1 [31] 'sffToCA' program (-trim chop -clear 454 -linker titanium -insertsize 3000 300). Reads were screened for vector and *E. coli *contamination using Seqclean http://compbio.dfci.harvard.edu/tgi/software. Next, reads were trimmed using Lucy ([49]; PindigoBAC536 (HindIII Splice): ≥ 50 bp). Randomly selected, processed reads in 5× coverage groups based on a 3 Mbp estimate were converted to FRG format with the CABOG script convert-fasta-to-v2.pl (paired: -454 -mean 3000 -stddev 20). FRG files were then assembled using various mixes of mated (PP), linear (L), or mate singleton (NM) reads into scaffolds with the wgs-assembler CABOG v6.1 http://wgs-assembler.sourceforge.net/; non-default parameters: overlapper = mer obtOverlapper = mer ovlOverlapper = mer unitigger = bog utgGenomeSize = 3000000 doToggle = 1). Scaffolds for all 19 assemblies were then ordered by BLASTN [36] alignment (E ≤ 1e-75; %Identity ≥ 98%) to FPC contig 23 MTP BAC end sequences and the expected position based on the MTP. Based on this order and orientation, a pseudomolecule was constructed for each assembly by concatenating the scaffolds with an insertion of 70 Ns between the scaffolds. No manual editing was performed at this stage. Pseudomolecules were then aligned, using BLASTN (version 2.2.15 with default parameters and no filtering; [36]), with the Sanger reference pseudomolecule and scored using a recently developed method [33]. Using this method, match score rewards for long contiguous matches with the reference and penalizes for assembly gaps, relocation score accounts for pairs of points that are in the correct order in the assembly with regard to the reference sequence, inversion score denotes the fraction of the assembly that is in the correct orientation relative to the reference, and coverage denotes the fraction of the reference that is covered by the assembly. The pseudomolecule derived from the 35L-20PP assembly was selected as the optimal assembly due to maximal coverage and match scores with regard to the reference Sanger pseudomolecule. Upon further inspection, it was discovered that two mis-selected MTP BACs were represented and that one BAC did not match BES and was not incorporated into the 20PP-35L pseudomolecule. Three MTP BACs (TCC_BB034K18; TCC_BB047M24; TCC_BB056I06) were therefore Sanger sequenced (as above), assembled as independent scaffolds, and built into a corrected version of the 20PP-35L pseudomolecule to replace two misplaced scaffolds. The MTP substitutions were as follows: TCC_BB034K18 replaced TCC_BB056D20, TCC_BB056I06 replaced TCC_BB056D12, and TCC_BB047M24 replaced TCC_BB070N13. The 20PP-35L pseudomolecule was then clipped of any remaining vector and *E. coli *contamination as identified by cross_match ([32]; -minmatch 10 -minscore 20 -screen). The final corrected pseudomolecule was validated by BLASTN [36] alignment (E < = 1e-75; %Identity > = 98%) of all the genetic marker sequences (available on request) in the region [29]. Comparative alignments were visualized using MUMMER software [35] or the LAGAN algorithm [50]. The sequence of the corrected pseudomolecule was deposited in GenBank under Accession #: JN127775.

### Functional profiling

The *T. cacao *unigene set (Build#2; 25,016 unique clusters) was downloaded from NCBI ftp://ftp.ncbi.nih.gov/repository/UniGene/Theobroma_cacao/. Unigene sequences were BLASTN-aligned to the 35L-20PP corrected pseudomolecule assembly (E ≤ 1e-75; T 5: additional file 6: Table S4). In this way, 249 unique unigene clusters were mapped to the pseudomolecule and these 249 unigenes were assigned functional annotations using Blast2GO software (Feb. 23 2011 build) [51] with 512 Mb RAM by first BLASTX-aligning (E ≤ 1e-6) them to the NCBI nr protein database (Feb. 23 2011 build) and then mapping to gene ontology (GO) terms in a local GO mapping database (Aug. 2010 build) as per the Blast2Go instructions http://www.blast2go.org/localgodb. Interproscan http://www.ebi.ac.uk/Tools/pfa/iprscan/ protein domain accession numbers (Release 31.0; Feb. 9 2011) were directly mapped to the unigenes with Blast2GO software using default parameters.

## Authors' contributions

FAF participated in experimental design, directed the project, constructed and analyzed the 454 assembly, and wrote the manuscript. CAS participated in experimental design, assembled a majority of the Sanger BACs and assisted in editing the manuscript. KM designed and directed the 454 sequencing experiments and assisted in editing the manuscript. ZS and JF prepared BAC pools and 454 sequencing libraries, carried out sequencing and assisted in editing the manuscript. NH and LP performed the scoring analysis. MES, SPF, and BPB were involved in MTP selection and assisted in editing the manuscript. CHC performed quality control analysis on BAC-ends. RS, DNK, and JCM participated in experimental design and assisted in editing the manuscript. All authors have read and approved the final manuscript.

## Supplementary Material

Additional file 1 **Assembly Statistics for Various Assembly Mixes**. Detailed assembly statistics for all CABOG assemblies. Click here for file

Additional file 2 **BAC End Sequence (BES) Position in Pseudomolecule Sorted by MTP Order**. BES BLASTN hit positions to 20PP-35L assembly. Click here for file

Additional file 3 **Where is the missing genome? **Potential unassembled 20PP-35L fractions of surrogate and degenerate contigs. Click here for file

Additional file 4 **Unmated Singletons plus Mate Pairs Can Substitute for Linear Reads**. Read mix assemblies (MUMMER plots) aligned to the Sanger reference pseudomolecule where reads came from true mate pairs (PP) or cases where a read had no matching mate (NM). Click here for file

Additional file 5 **Individual Sanger Sequenced BACs aligned to 454 pseudomolecule**. MUMMER plots of individual Sanger-sequenced BAC assemblies mostly match with corrected 35L-15PP 454 pseudomolecule. Click here for file

Additional file 6 **Pseudomolecule Unigene Hit Position**. BLASTN hits of unigenes to 20PP-35L pseudomolecule. Click here for file

Additional file 7 **Pseudomolecule Unigene Annotation**. One row per gene annotation of the unigenes that hit the 20PP-35L pseudomolecule. Click here for file

Additional file 8 **Pseudomolecule Unigene Annotation (Database-Friendly)**. One row per annotation of the unigenes that hit the 20PP-35L pseudomolecule. Click here for file

Additional file 9 **249 unigene sequences in FASTA format**. Unigene cDNA sequences localized to contig assembly. Click here for file
