# Supplementary material for: Sequencing of a QTL-rich region of the Theobroma cacao genome using pooled BACs and the identification of trait specific candidate genes
Source: BMC Genomics. 2011 Jul 27;12:379. doi: 10.1186/1471-2164-12-379 (PMC3154204; doi:10.1186/1471-2164-12-379)
Supplement: Additional file 4 — Unmated Singletons plus Mate Pairs Can Substitute for Linear Reads. Read mix assemblies (MUMMER plots) aligned to the Sanger reference pseudomolecule where reads came from true mate pairs (PP) or cases where a read had no matching mate (NM). [file 1471-2164-12-379-S4.PPT]

## Slide 1
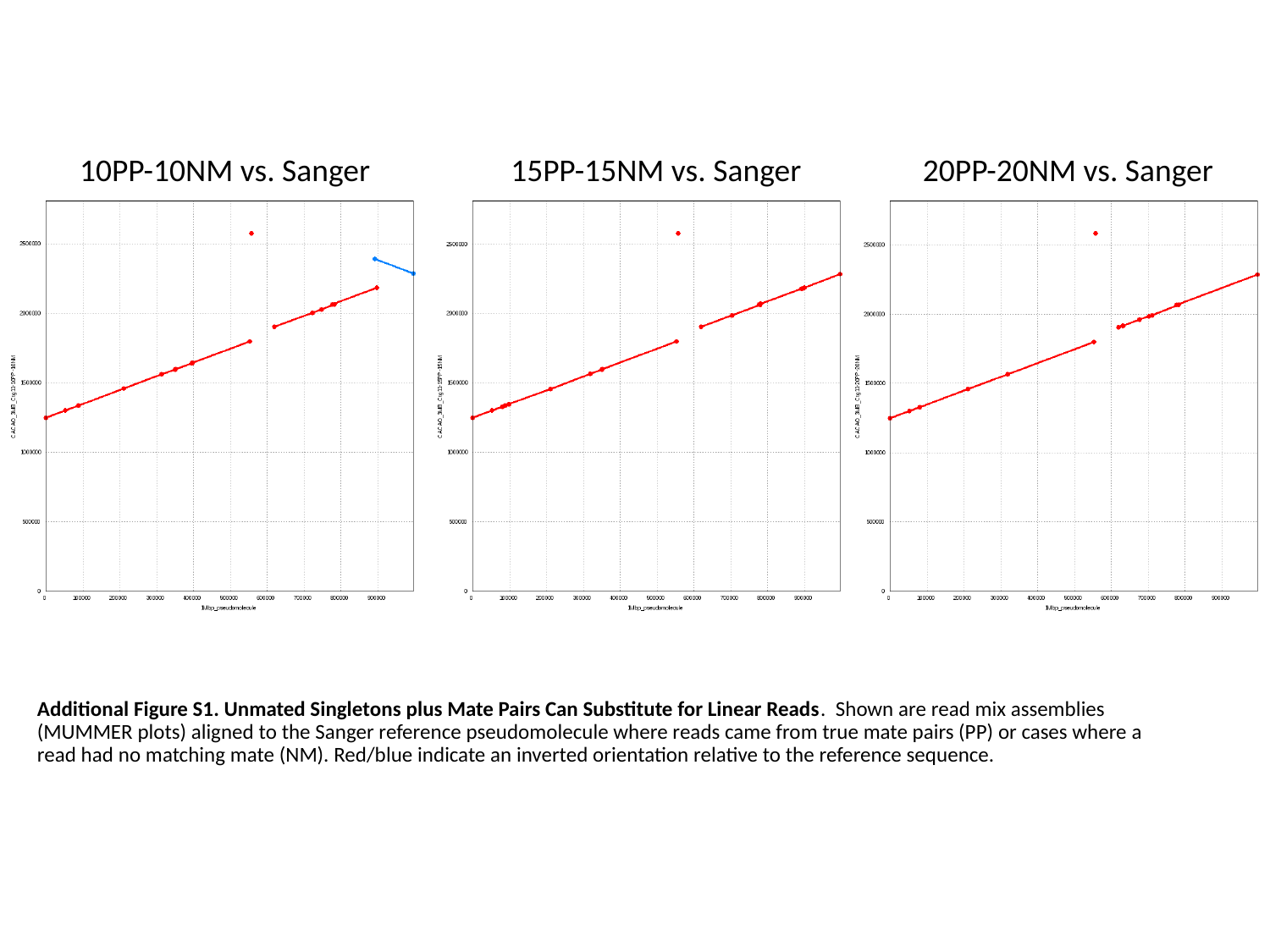

10PP-10NM vs. Sanger
15PP-15NM vs. Sanger
20PP-20NM vs. Sanger
Additional Figure S1. Unmated Singletons plus Mate Pairs Can Substitute for Linear Reads. Shown are read mix assemblies (MUMMER plots) aligned to the Sanger reference pseudomolecule where reads came from true mate pairs (PP) or cases where a read had no matching mate (NM). Red/blue indicate an inverted orientation relative to the reference sequence.
