# Supplementary material for: Sequencing of a QTL-rich region of the Theobroma cacao genome using pooled BACs and the identification of trait specific candidate genes
Source: BMC Genomics. 2011 Jul 27;12:379. doi: 10.1186/1471-2164-12-379 (PMC3154204; doi:10.1186/1471-2164-12-379)
Supplement: Additional file 5 — Individual Sanger Sequenced BACs aligned to 454 pseudomolecule. MUMMER plots of individual Sanger-sequenced BAC assemblies mostly match with corrected 35L-15PP 454 pseudomolecule. [file 1471-2164-12-379-S5.PPT]

## Slide 1
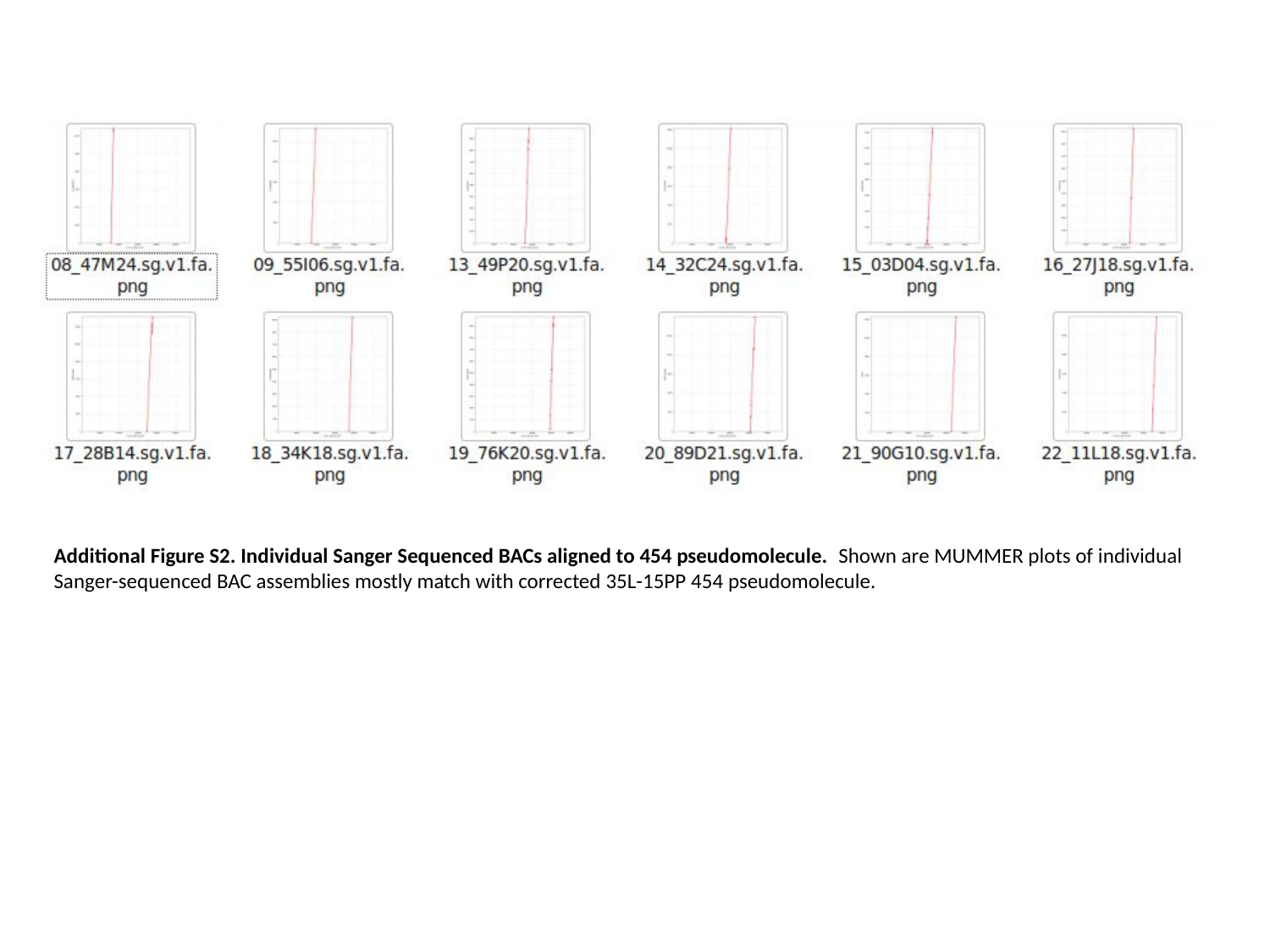

Additional Figure S2. Individual Sanger Sequenced BACs aligned to 454 pseudomolecule. Shown are MUMMER plots of individual Sanger-sequenced BAC assemblies mostly match with corrected 35L-15PP 454 pseudomolecule.
